# Mental health professionals’ perspectives on the relevance of religion and spirituality to mental health care

**DOI:** 10.1186/s40359-023-01466-y

**Published:** 2023-12-12

**Authors:** Cassandra Vieten, Holly K. Oxhandler, Michelle Pearce, Nina Fry, Chloe Tanega, Kenneth Pargament

**Affiliations:** 1 Clarke Center for Human Imagination, Department of Physical Sciences, University of California, La Jolla, San Diego, CA USA; 2 Baylor University, Waco, TX USA; 3 University of Maryland, Baltimore, MD USA; 4 Institute of Noetic Sciences, Novato, CA USA; 5 Bowling Green State University, Bowling Green, OH USA

**Keywords:** Spirituality, Religion, Mental health services, Cultural competency, Religion and psychology

## Abstract

**Background:**

A large body of evidence indicates that spiritual and religious backgrounds, beliefs, and practices (SRBBPs) are related to better psychological health. Spirituality and religion (R/S) are also important aspects of multicultural diversity. There is evidence that clients would like to talk about their spirituality, and that including it in assessment and treatment planning can be beneficial. However, the extent to which practicing mental health professionals view SRBBPs as relevant to mental health and clinical practice is unclear.

**Methods:**

A survey examining several aspects of addressing SRBBPs in clinical practice was distributed to 894 professionals across mental health disciplines, including psychiatry, psychology, social work, marriage family therapy, licensed professional counselors, certified chemical dependency counselors, and psychiatric mental health nurses.

**Results:**

89% of mental health professionals agreed that clinicians should receive training in R/S competencies. There were no differences between mental health disciplines in ratings of importance of such training. Younger individuals and those who identify as more spiritual were more likely to consider R/S training as important. Although 47.1% of professionals had not received much R/S training, many perceived themselves to be highly competent in R/S clinical integration practices (57.8% considered themselves able to display them very much or completely). In addition, respondents with more R/S training evaluated themselves as more proficient in R/S clinical integration. Nearly two-thirds (65.2%) of respondents reported encountering few to no barriers to engaging in R/S competent mental health care.

**Conclusions:**

There is a growing consensus among mental health care professionals that mental health professionals should be trained in R/S competencies. Strong agreement exists that basic R/S competencies include respect, empathy, examination of bias, and routine assessment of R/S in mental health care. Four in five of those surveyed agree that more active competencies, such as identifying and addressing religious and spiritual struggles and problems and helping clients explore and access R/S strengths and resources should be included, whereas one in five report less comfort with these competencies.

**Supplementary Information:**

The online version contains supplementary material available at 10.1186/s40359-023-01466-y.

## Background

Gallup polls between 1992 and 2021 show that despite a substantial decline over time, 72% of Americans still report that religion is “very important” or “fairly important” in their lives [1]. Over 80% of people believe in God [2], 75% of people report praying to God “often” or “sometimes” [1], and 41% of US citizens attend a church or synagogue [1]. A Pew Forum survey [3] indicates that 27% of people self-identify as “spiritual, but not religious,” compared with 19% in 2012. Still, two-thirds of those say they believe in God (68%), and one-in-five (21%) say they pray every day [3].

A large body of evidence indicates that spiritual and religious backgrounds, beliefs, and practices (SRBBPs) are relevant to most people’s psychological well-being [4]. Involvement in religious and spiritual (R/S) practices and communities is related to lower depression, anxiety, suicide ideation and attempts, post-traumatic stress disorder (PTSD), and substance abuse, as well as a higher purpose in life, hope, optimism, and self-esteem [5, 6]. SRBBPs can serve as a vital resource for people, with adaptive benefits such as self-regulation, positive attachment, emotional comfort, meaning, and spirituality [7]. Spiritual transcendence has been identified as one of the most important needs in aging populations [8]. A recent nationwide survey of 989 mental health care clients showed that 64.9% agreed that engaging in SRBBPs “improves my mental health,” and 64% viewed their R/S as relevant to their mental health [9]. In a recent survey of 2,050 individuals receiving mental health services and their family members, 80% agreed or strongly agreed that spirituality was important to their mental health [10]. Evidence shows that spiritual and religious coping is prevalent and beneficial for combat veterans [6], disaster survivors [11], recovery from interpersonal trauma [12], and multiple mental health and quality of life outcomes [13].

Engagement in SRBBPs has also been linked to psychological and emotional difficulties and disorders. R/S struggles (or tension, strain, and conflict around sacred matters within oneself, with others, or with the supernatural) have been linked with depression, paranoid ideation, somatization, anxiety, PTSD, social isolation, and lower life satisfaction [14]. R/S struggles have also been associated with immune system declines, slower rehabilitation from disease, declines in emotional and physical health, and mortality [15]. Trauma or distress arising from abuse by clergy, or rejection by or alienation from R/S organizations or communities due to religious ideology, gender discrimination, sexual orientation, divorce, or abortion are underexplored potential causes of psychological distress. Since 1994, “Religious or Spiritual Problem” (DSM-5; Code V62.89; ICD-10 Code 65.8) has been included in the *Diagnostic and Statistical Manual of Mental Disorders* [16, 17] and *ICD-10* [18]. Religious problems can be a byproduct of psychiatric illness, a source of psychiatric illness, or a combination of both [7].

Spirituality and religion are aspects of multicultural diversity [19, 20], overlapping with race, ethnicity and culture, and intersecting with other elements of diversity such as gender and sexual orientation. Like other forms of diversity, religious persecution and discrimination based on religious or spiritual beliefs have historically been and continue to be widespread [21]. Professional and accrediting organizations across mental health professions have now included religion (and sometimes spirituality) in their definitions of culture or diversity. Training in cultural competencies are required across mental health professions’ codes of ethics, and in healthcare practice guidelines for assessment and treatment [22]. However, most required training in multicultural competency focuses on ethnic and racial diversity, and training programs pay inadequate attention to religious and spiritual aspects of diversity in multicultural training [23, 24].

There is evidence that clients in mental health care see their SRBBPs as relevant to their psychological well-being [25]. Over 60% of mental health clients agree or strongly agree to items, such as “Relying on my religious/spiritual beliefs helps me to feel mentally healthy” and “My religious/spiritual beliefs help me to reach my mental health potential” [9]. Clients also largely agree that “It is important for my therapist to know how to discuss my religion/spirituality in mental health therapy” (58.9%) [26]. Most clients (51.2%) reportedly agree that discussing their RS beliefs in treatment improves their mental health outcomes [9].

Mental health care providers in large part agree that SRBBPs are relevant to mental health. Upon reviewing 16 proposed aspects of basic RS competency, between 73 and 94% of psychologists agree (depending on which specific competency) that psychologists should receive training and be required to demonstrate competence [27]. However, 52–81% reported having received little or no training, and 30–59% reportedly receiving no training at all [27]. Another survey of 543 psychology doctoral students indicated that most received no formal training in SRBBPs, and almost universally endorsed the idea that patients should be asked about SRBBPs [28]. Likewise, a nationwide survey of social workers shows a strong majority held high levels of self-efficacy and positive attitudes toward integrating clients’ RS into treatment, but fewer actually do so [29], in part due to lack of training.

To remedy this lack of training, proposed competencies have been developed [30], and initial work indicates that these are acceptable to psychologists [27]. In addition, an online training has been developed and shown to be feasible, acceptable, and to increase mental health professionals’ self-reported competence and content knowledge [31, 32]. With notable exceptions [33, 34], few surveys have been conducted across mental health professions nationwide to determine how relevant providers’ believe SRBBPs are to mental health care, which competencies should be included in training, and which competencies they have received training in and currently employ.

### Current study

This project focused on understanding how practicing mental health professionals (N = 894) across six professions view the relevance of spirituality and religion to mental health and clinical practice. We examined their views about the need for training in R/S competencies and the extent to which they include them in their own clinical practice. We also investigated how those perceptions are influenced by their profession, level of training, and their own SRBBPs.

The study was pre-registered with Open Science Framework on August 2, 2018. We hypothesized, based on results of previous surveys [27, 30], that H_1_) over 70% of mental health professionals would agree that licensed and practicing clinicians should receive training in R/S competencies; H_2_) over 50% of mental health professionals would report receiving little to no explicit training in R/S competencies; H_3_) over 50% of mental health professionals would rate themselves as mostly or completely competent in addressing their clients SRBBPs, despite a general lack of training; H_4_) mental health professionals would report at least one barrier to engaging in spiritually competent mental health care; H_5_) higher levels of training in R/S in mental health care would be associated with higher levels of perceived competency; and H_6_) mental health providers who are older and currently identify with a R/S orientation would score higher in their perceptions of the importance of training in religious and spiritual competencies.

## Methods

### Participants

This study was reviewed and considered exempt by the Institutional Review Board of the Institute of Noetic Sciences. Licensed, certified, and practicing mental health professionals from psychiatry, psychology, social work, marriage family therapy, professional counseling, and pastoral counseling were recruited in the United States between October 2018 and May 2019. Psychiatric nurses were added to the list of professions after several responded and indicated being a licensed mental health care provider. Participants were recruited from list-servs and multiple Facebook groups for mental health professionals. Materials were also distributed at state-level and national professional conventions, including email lists from multiple state licensing boards of mental health professionals.

To reduce selection bias, recruitment materials did not indicate that the survey was focused on R/S, but instead invited participants to participate in a survey about “views on training for specific competencies in mental health care.” Participants were not reimbursed, but instead offered the opportunity to be entered into a drawing to receive one of four Apple iPads. Four participants received an Apple iPad after all data were collected. Responses to the survey were collected anonymously online, using Survey Monkey. Contact information was optionally collected in a separate, unrelated survey from participants who opted into the iPad drawing or wished to receive results of the study.

### Procedures

Consent was obtained on the first page of the online survey. Participants were excluded if they reported not being licensed or certified, or not actively providing mental health care services. Participants then responded to survey items which took between 25 and 35 min to complete.

### Measures

Demographics were collected including age, gender, income, race/ethnicity, relationship status, and region of residence. Variables related to professional training and practice included level of education, degree obtained, license obtained, years in practice, specialty, client load, primary setting, and training in spirituality and religion.

The survey provided working definitions of spirituality and religion, mental health, and mental illness (See Additional File 1). Providers were asked about their views on the relevance of R/S to mental health and clinical practice using five items mirroring the Religious/Spiritually Integrated Practice Assessment Scale – Client Attitudes (RSIPAS-CA), which has shown very good validity and reliability (α = 0.89) [35]). In addition, we included investigator-developed items related to perceived barriers to assessing or attending to R/S in clinical practice (such as, *not enough time* or *I don’t think my clients or patients would appreciate it*). The extent to which providers reported engaging in R/S assessment or discussion in their actual clinical practice was assessed using the “Frequency of Engaging in R/S Integrated Practice” subscale of the Religious/Spiritually Integrated Practice Assessment Scale (RSIPAS) [33, 36], which demonstrates excellent reliability and validity (α = 0.95) .

Participants rated whether they agreed that (1) mental health care professionals should receive training in R/S competencies, (2) whether they received training, and (3) self-rated their competence in 15 of the 16 competency areas identified by Vieten et al. [27, 30]. One competency regarding staying abreast of the scientific literature on the relationship between R/S and mental health was removed due to receiving the lowest endorsement in two previous studies of psychologists, indicating most people in the field see this as exceeding a basic level of R/S competence.

Open-ended items included questions about perceived barriers to including clients’ R/S into practice, anything that has helped or supported to them assess clients’ R/S into clinical practice, and anything they have done regarding assessing or including clients’ R/S that they found to be particularly helpful or supportive.

Finally, SRBBPs were measured using the Intrinsic Religiosity scale of Duke University Religious Index (DUREL) (α = 0.89) [37], which was modified to include “spirituality” as well as religiosity. We also included items on religious and spiritual identity from the Brief Multidimensional Measure of Religiousness/Spirituality (BMMRS) (α = 0.70) [38]. We asked questions regarding current religious affiliation, how liberal or conservative respondents perceived their religious/spiritual beliefs to be, how much R/S influenced respondents’ lives growing up, and frequency of private and external R/S activities. These questions were intentionally placed at the end of the survey to reduce potential priming.

## Results

A total of 1,327 people visited the survey landing page. Thirteen participants did not proceed beyond this page, and another 49 did not proceed beyond the online consent form, which revealed the topic of the survey. An additional 34 did not complete the survey, and 3 completed the survey, but reported not being licensed or certified mental health professionals, leaving a total of 1,241 participants included in the sample.

### Sampling bias

Upon preparing the data for analysis, we recognized that nearly half of the respondents who had taken degree courses or continuing education workshops in R/S had attended more than three courses (*n* = 144, 41.5%) or continuing education workshops (*n* = 265, 50.8%). This is an unusually high level of training in R/S when compared with previous data on prevalence of training among mental health professionals [27, 29]. While this could reflect recent increases in R/S training across mental health professions, sampling bias is more likely. Despite our efforts to reduce biased sampling by masking the topic of the survey and using recruitment sources without an R/S focus, the recruited sample appeared to include a higher number than usual number of mental health professionals with a strong interest in R/S.

Since our intention was to gain input from a more generalized sample of mental health professionals, to be conservative and avoid results and interpretations with a positive bias toward R/S, we elected to remove data from respondents who had attended more than 3 courses in R/S (*n* = 86), as well as participants who were pastoral counselors (*n* = 79). Finally, data from 19 participants who were not mental health providers was deleted. In total, 894 participants were included in the analyses.

### Demographic characteristics

The majority of participants were women (78.0%), aged either between 45 and 54 years (25.0%) or between 55 and 64 years (26.4%). Most respondents were White (79.6%), married (61.0%), and lived in suburban areas (61.3%). Less than half of respondents made over $100,000 annually (43.7%) (See all demographic information in Additional File 2).

### Professional background and training

The majority of respondents had a master’s degree (67.6%), followed by those with a doctoral degree (31.4%). 28% were licensed social workers, 21.9% were licensed professional counselors, 19.1% were licensed marriage family therapists, 14.4% were licensed clinical psychologists, 4.4% were psychiatrists, 2.0% were chemical dependency counselors, and 0.8% were psychiatric mental health nurses. Most respondents worked either in solo private practice (40.2%) or at a non-profit agency (20.6%). Professionals reported a mean of 19.29 (*SD* = 11.17) years in clinical practice (See all educational and professional background in Additional File 3).

### Spiritual and religious background, beliefs and practices

Nearly half the sample (41.1%) was Christian, while 35.1% considered themselves spiritual but not religious. 63.7% reported they were either not religious or only slightly religious, whilst 48.4% described themselves as very spiritual.Most participants (55.9%) considered their R/S to be liberal, 17% moderate, and 17.6% conservative. Finally, high levels of daily spiritual experiences were observed, including 71.7% experiencing the presence of the divine (71.7%), R/S lying behind their approach to life (71.7%), and trying to carry R/S into all areas of life (65.4%) (See all R/S backgrounds, beliefs and practices in Additional File 4).

### Hypothesis testing

#### H_1_ views on training in R/S competencies

It was hypothesized that 70% of mental health professionals would agree that licensed and practicing clinicians should receive training in R/S competencies. Across all competencies, 89.1% of the respondents agreed *somewhat* or *very much* and 10.9% agreed a little bit or not at all. Depending on the specific competency, between 81.2% and 96.0% of all participants endorsed that mental health professionals should receive explicit training *somewhat* or *very much*, and over 50% of participants endorsed “*very much*” for all competencies. Therefore, H_1_ was supported.

Mental health professionals displayed the greatest levels of agreement with the training on demonstrating empathy, respect, and appreciation to R/S diverse clients (96.0%), conducting empathic and effective psychotherapy with R/S diverse clients (94.9%) and cultivating awareness of clinicians’ R/S influence on psychological processes (94.8%). The lowest levels of endorsement were observed in the training on identifying and addressing R/S problems in clinical practice (81.2%), helping clients explore and access R/S strengths and resources (83.0%) and differentiating between spirituality and religion (84.8%) (See all descriptive analysis in Additional File 5).

#### H_2_ amount of explicit R/S competency training

It was hypothesized that 50% of mental health professionals would report receiving little to no explicit training in religious and spiritual competencies. Nearly half of the respondentsindicated that during their professional degree program they had not received anyor not very much training in addressing R/S in practice. (Table 1).

**Table 1:** Frequency analysis on training in R/S competencies

	*n*	%
Did you receive any training or education on R/S as a form of multicultural diversity that you might encounter in your clients?		
**R/S education during professional degree program**		
None	168	18.9
Not very much	251	28.2
Some	312	35.1
Quite a bit	124	13.9
A lot	34	3.8
**R/S courses during degree program**		
No course, did not receive any information	233	26.2
No course, but received some information	503	56.5
Yes	155	17.4
**Continuing R/S education workshop/courses**		
No	636	71.4
Yes	255	28.6
**Other training**		
Reading books or articles	617	69.0
Attending conference presentations	265	29.6
Clinical supervision/consultation	297	33.2
Conversations	537	60.1
Retreats	140	15.7
Personal exploration	551	61.6

Across all R/S competencies, 69.4% reported no training at all or a little bit of training. Consequently, H_2_ was supported. The least training was observed in identifying of potentially harmful R/S practice, beliefs, experiences; being aware of R/S resources and practices supporting mental health; and identifying and addressing R/S problems in clinical practice. The highest levels of training (nearly 50% reported some training) were reported for awareness of clinicians’ R/S influencing their views on psychological processes, conducting empathic and effective psychotherapy with R/S diverse clients, and understanding of R/S importance to human diversity (See descriptive analysis on explicit R/S competency traning in Additional File 6).

#### H_3_ self-rated R/S competence

##### Hypothesis 3

postulated that 50% of mental health professionals would rate themselves as mostly or completely competent in R/S domains, despite a general lack of training. Half of the respondents (49.9%) rated themselves as having *quite a bit* or *a lot of* proficiency in attending or integrating clients’ or patients’ R/S backgrounds, beliefs, and practices in mental health care. Across all R/S competencies, depending on the particular competency, 57.8% indicated that they were able to display them *very much* or *completely*. Therefore, H_3_ was supported.

The least proficiency in self-rated R/S competencies were awareness of R/S legal and ethical issues related to clinical practice (37.0%), identification of potentially harmful R/S practice, beliefs, experiences (44.3%), and identification and address of R/S problems in clinical practice (42.9%). The highest self-rated R/S competences were awareness of clinicians’ R/S influence on psychological processes (74.8%), empathy, respect, and appreciation to R/S diverse clients (75.1%) and understanding of R/S importance to human diversity (73.7%) (See descriptive analysis on self-rated R/S competence in Additional Files 7).

#### H_4_ barriers to R/S competent mental health care

It was hypothesized that mental health professionals would report at least one barrier to engaging in R/S competent mental health care. In total, nearly two-thirds (65.2%) of respondents reported that *nothing makes it less likely* that they would attend to R/S in clinical practice. In the remaining 34.8% who did perceive at least one barrier, 11.1% did not have enough time, 8.9% felt they did not have enough training in it, 6.7% stated that their institution/setting does not support it, 5.9% thought their clients would not appreciate it, 2.3% felt personally uncomfortable doing so, 1.6% thought it was not important, 1.3% thought R/S issues should not be discussed in clinical work, and 8.9% cited other barriers. Respondents who chose the other option mostly reported that their engagement would depend on the particular client since R/S issues are seen as a sensitive topic that should be brought up by the client first or once a therapeutic alliance has been established. Consequently, H_4_ was not supported.

#### H_5_ R/S training as a positive predictor of self-rated R/S proficiency

A simple linear regression analysis was conducted to evaluate if R/S training was a statistically significant positive predictor of R/S self-rated proficiency. A total score was computed for the number of R/S classes and continuing education/workshops attended. Self-rated proficiency was rated with a single item (“Please rate how much proficiency you have in attending to or integrating your clients’ or patients’ R/S backgrounds, beliefs and practices in mental health care”) with higher scores indicating greater self-rated proficiency.

The analysis indicated a model that was statistically significant [*F*(1, 325) = 4.61, *p* = .032], but accounted for only 1.1% of the variance in self-rated proficiency (*R*^2^ = 0.014, *R*^2^_adj_. = 0.011). R/S training was a also statistically significant positive predictor of perceived competency (β = 0.12 *t* = 2.15, *p* = .032), suggesting that individuals who attended a greater number of R/S classes and continuing education/workshops were more likely to evaluate themselves as more proficient in R/S clinical integration. Therefore, H_5_ was supported.

#### H_6_ age and R/S orientation as positive predictors of importance of R/S training

A multiple linear regression was performed to examine if age and R/S orientation were statistically significant positive predictors of importance of R/S training. A total mean score to assess importance of R/S training across all R/S competencies was calculated, with higher scores being indicative of greater importance. A statistically significant model was identified that accounted for 6.9% of the variance in importance of R/S training.Age was a statistically significant negative predictor, whereas spiritual orientation was a statistically significant positive predictor, suggesting that younger and more spiritual individuals were more likely to consider R/S training more important. Religious orientation was not a statistically significant predictor (Table 2). Consequently, H_6_ was partially supported.

**Table 2:** Multiple linear regression with importance of R/S training as the outcome variable and age and R/S orientation as the predictor variables (*N* = 875)

	*B* (95% CI)	*SE*_*B*_	β	*t*	*p*
**Variable**					
(Constant)	3.06(2.91, 3.21)	0.077		39.95	< 0.001
Age	− 0.028(-0.055, − 0.001)	0.014	− 0.068	-2.02	0.044
Religious orientation	− 0.003(-0.038, 0.033)	0.018	− 0.005	− 0.16	0.87
Spiritual orientation	0.17(0.13, 0.22)	0.022	0.28	7.82	< 0.001

*Note*. *B* = unstandardized regression coefficient; *CI* = confidence interval; *SEB =* standard error of unstandardized regression coefficient; β = standardized regression coefficient

### Secondary analyses

#### Differences between professional disciplines

ANOVAs were conducted to assess whether there were differences across mental health disciplines (psychiatrists, psychologists, MFT, LCSW, professional counselors, and pastoral counselors) in their rating of importance of R/S competencies in training, self-rated R/S competence, and having received training in R/S competence between. There were no significant differences between disciplines, with the exception of pastoral counselors reporting having received more training in R/S competencies than other professions F(5,661) = 3.67,p = .003.

#### R/S inquiry and engagement

On average, mental health professionals reported verbally inquiring about religion or spirituality in the course of assessment or treatment with over 60% of their clients. Just over half of respondentsinquired with three-quarters or more of their clients/patients, about a fifth inquired with almost all of their clients/patients, and almost a third inquired with less than about a third of clients/patients. Regarding actual engagement in other clinical practices addressing R/S, nearly two-thirds of mental health professionals reported engaging *very often* or *often* in helping clients consider ways their R/S support systems may be helpful, just over half reported both involving clients in deciding about R/S treatment integration and helping clients consider R/S meaning and purpose of current life situations.(Table 3).

**Table 3:** Descriptive analysis on engagement in R/S integrated clinical practice

	*M*	*SD*	% Often	% Very often	% Often + Very often
**How frequently you actually have done each of the following in your clinical practice?**					
1. Use of R/S empirically supported interventions	2.30	1.15	10.2	4.9	15.1
2. Seek out R/S consultation	2.49	0.92	9.8	2.1	11.9
3. Read R/S research on mental health to guide practice decisions	2.60	1.07	14.6	4.5	19.1
4. Read about integration ways of clients’ R/S to guide practice decisions	2.77	1.03	17.5	5.1	22.6
5. Link clients with potentially helpful R/S resources	3.28	1.18	27.4	16.9	44.3
6. Conduct full bio-psycho-social-spiritual assessment with clients	3.28	1.46	19.0	29.7	48.7
7. Help clients consider R/S meaning and purpose of current life situations	3.44	1.13	32.1	18.9	51.0
8. Involve clients in deciding about R/S treatment integration	3.50	1.21	25.3	26.0	51.3
9. Help clients consider ways their R/S support systems may be helpful	3.75	0.96	40.2	23.2	63.4

*Note*. Items appear in ascending order based on mean scores. Item values correspond to 1 = Never, 2 = Rarely, 3 = Some of the time, 4 = Often, 5 = Very often

#### Perceived importance of R/S training and sociodemographic characteristics

A multiple linear regression was performed to investigate if gender, age, R/S training, R/S orientation, frequency of attendance at religious services (ORA), frequency of private religious activities (NORA), intrinsic religiosity (IR), and R/S upbringing would be statistically significant predictors of importance of R/S training. A total score was computed for the number of R/S classes and continuing education/workshops attended. Regarding IR, a mean score was calculated for the three items included in the subscale, with higher scores indicating greater IR. Finally, a mean score was calculated for the 15 items training importance in specific R/S competencies, with higher scores suggesting greater perceived importance.

The analysis indicated a statistically significant model that accounted for 9.9% of the variance in importance of R/S training. Gender, R/S training, and spiritual orientation were statistically significant positive predictors, suggesting that women and professionals with greater R/S training and spiritual orientation were more likely to perceive R/S training as more important. The rest of the predictors were notstatistically significant (Table 4).

**Table 4:** Multiple linear regression with importance of R/S training as the outcome variable and gender, age, R/S training, R/S orientation, frequency of attendance at religious services (ORA), frequency of private religious activities (NORA), intrinsic religiosity (IR), and R/S upbringing as the predictor variables (*N* = 314)

	*B* (95% CI)	*SE*_*B*_	β	*t*	*p*
**Variable**					
(Constant)	2.69 (2.39, 3.00)	0.15		17.52	< 0.001
Gender	0.16 (0.027, 0.30)	0.068	0.13	2.36	0.019
Age	0.013 (-0.031, 0.057)	0.022	0.033	0.586	0.56
R/S training	0.064 (0.018, 0.11)	0.023	0.153	2.75	0.006
Religious orientation	− 0.018 (-0.083, 0.048)	0.033	− 0.037	− 0.53	0.60
Spiritual orientation	0.12 (0.028, 0.21)	0.045	0.19	2.60	0.010
R/S Upbringing	0.007 (-0.028, 0.042)	0.018	0.021	0.38	0.71
ORA	− 0.011 (-0.056, 0.035)	0.023	− 0.032	− 0.46	0.64
NORA	0.013 (-0.028, 0.055)	0.021	0.043	0.63	0.53
IR	0.049 (-0.051, 0.15)	0.051	0.078	0.96	0.34

*Note*. ^a^ 0 = male, 1 = female. *B* = unstandardized regression coefficient; *CI* = confidence interval; *SEB =* standard error of unstandardized regression coefficient; β = standardized regression coefficient

## Discussion

In this study, 894 professionals practicing across mental health disciplines (counseling, social work, marriage family therapy, psychology, psychiatry/psychiatric nursing) were asked to complete questionnaires assessing their views on addressing SRBBPs in clinical practice, their own training in this area, and how they engaged with R/S in practice. The overwhelming majority (89.1%) of mental health providers agreed that licensed and practicing clinicians should receive training in spiritual and religious competencies. There were no differences between mental health disciplines in ratings of importance of such training. Most reported little or no training in how to ethically and effectively address R/S in their practice of mental health careand over a quarter receiving no coursework or information during their graduate training. Despite low levels of training, over half report having high levels of proficiency in attending or integrating R/S. Very few mental health professionals report encountering barriers to addressing R/S in their practice. Secondary analyses showed that personal spirituality, being younger, being female, and having received training in R/S competencies were associated with viewing R/S competencies as more important.

### A growing consensus

Our finding of widespread affirmation of the importance of, and need for training in, R/S competencies across mental health professions mirror previous surveys in individual professions (e.g. psychology [27], social work [29], medicine [39]). This growing body of research indicates that there is some consensus across mental health professions that training in R/S competencies is appropriate for providers of mental health services.

In fact, in this sample, higher numbers of people report having received some training in R/S compared to previous research, and practicing clinicians report fewer barriers to integrating R/S competence into their clinical settings. Mental health professionals in our sample reported verbally inquiring about religion or spirituality in the course of assessment with the majority of their clients, and more frequently integrating R/S into treatment than in previous surveys (e.g. psychology [27], social work [29], interdisciplinary [34]). The most frequent ways respondents reported engaging in actual practice were (1) helping clients consider ways their R/S support systems may be helpful, (2) involving them in deciding about R/S treatment integration, and (3) helping them consider the R/S meaning and purpose of their current life situations. It is possible that the field is already changing to more frequently include R/S in training and assessment.

This may be a result of increased attention to diversity, equity and inclusion in general. It could also be in part explained by the increasing evidence-base for the intersection of R/S and mental health, or greater awareness of this evidence-base. This might also reflect the growth in resources that describe how practitioners can address spirituality in psychotherapy [40–42]. The popularity and growing empirical support for third-wave therapies such as dialectic behavior therapy and acceptance and commitment therapy, which include (primarily secularized) elements of contemplative practices and theories, could also be partially responsible for increased attention to, and fewer barriers to, addressing spirituality in mental health care. In addition, our data show that, not surprisingly, mental health professionals who identify as more spiritual are more likely to endorse these competencies, which has been noted in previous studies and as part of Namaste Theory [43]. Further, those who are younger are more likely to endorse these competencies, which could indicate that people newer to the field and have more multicultural perspectives are more likely to view these competencies as important.

### Lower support for more active R/S competencies

Most existing frameworks for R/S competency expect competent clinicians to not only respect their clients’ or patients’ SRBBPs and not discriminate based on them, but to actively inquire about them, and assist them in accessing R/S strengths or navigate R/S struggles. However, participants in this survey ranked training in (1) helping people access R/S strengths and resources and (2) being able to help with R/S problems or struggles among the lowest inimportance (though still over half viewing their importance as “very much”).

This may mean that many mental health professionals consider these competencies too advanced to be considered general competence, and that training in them should be reserved for people who specialize in R/S and mental health. It could also mean that the scholars and researchers who develop such guidelines (and presumably have greater expertise in multicultural diversity or in the intersection of R/S and are familiar with the research demonstrating the links between R/S and mental health) are justifiably encouraging the field to reach a higher level of competence in these more active clinical behaviors. This has been the case with other arenas of competency, where the consensus of the field may have initially found it unnecessary to attend as closely to, for example, LGBTQ + diversity, until it was better understood how sexual orientation impacts psychological functioning and therapeutic alliance.

This lower ranking could also mean that the general population of mental health providers do not possess the knowledge, skills, or experience to identify and address R/S struggles or to help clients access R/S resources, and therefore feel less confident in endorsing their inclusion in training or treatment. Indeed, these were among the lowest in mental health providers’ self-rated competence. Data from this survey and other work [27, 29, 31] show that receiving training in R/S competence increases self-rated comfort and confidence in employing these skills.

Finally, it could be that these more active competencies could be reworded to gain greater consensus. For example, over half of the mental health professionals in this survey reported engaging *very often* or *often* in “helping clients consider ways their R/S support systems may be helpful.” This more reflective language found in the behaviors subscale of the RSIPAS [36] may be better suited to gaining consensus.

### Limitations

This was a cross-sectional survey that did not rely on random selection. Interpretation involving causation or generalizability should be avoided or made with caution. While efforts were made to reduce sampling bias by masking the topic of the survey and avoiding R/S related recruitment sites and list-servs, it is possible that the sample is non-representative of mental health professionals. A greater number of respondents replied that they had received more training in R/S than previous research indicated, and outliers were removed to be conservative and not overestimate the level of endorsement of R/S competencies. The greater prevalence of training in relation to previous research could also be explained by the sample being cross-disciplinary and weighted toward professional counselors, many of whom were substance abuse counselors who tend to have more training in R/S or spiritually-oriented (e.g. 12-step) interventions. Future studies should also identify how these findings might vary across mental health disciplines.

## Conclusions

The results of this study confirm and extend previous findings indicating that there is a strong consensus among mental health care professionals that mental health professionals should be trained in R/S competencies. There is agreement that basic R/S competencies include respect, empathy, examination of bias, and routine assessment of R/S in mental health care. Four in five of those surveyed agree that more active competencies, such as identifying and addressing R/S struggles and helping clients explore and access R/S strengths and resources should be included.

This cross-disciplinary survey is another important step in carefully determining whether and how religion and spirituality should be attended to in mental health care, as well as how mental health professionals should be trained in R/S competencies. This sample reported greater levels of training and integration into practice, with fewer perceived barriers, than has been reported in previous work. More training appears to lead to greater self-rated competence and actual practice behaviors. Yet still, nearly 79% report no or very little training in this area.

To address this gap, our team is working next on both practical guidelines for both teaching R/S competencies, as well as professional practice guidelines for ethically attending to R/S in the practice of mental health care. The teaching guidelines in development include suggested syllabi and/or content domains to be sure to cover in stand-alone curricula (such as a workshop or course), as well ways to infuse R/S competencies into other courses. For example, a multicultural competency course could include religious and spiritual beliefs and practices in multicutural case studies, or a clinical practice course could provide opportunities to practice skills for inquiring about R/S along with other forms of diversity and functional domains like work, relationships, or health. In clinical practice, clinicians can begin to routinely include inquiring about R/S in initial assessments and taking a client history, as well as asking about whether religious or spiritual activities have been useful coping strategies.

Our hope is that attending to the R/S dimensions of clients’ lives will elevate the effectiveness of mental health care across discplines, and help to reduce disparities in access to and utilization of mental health care in the majority of people for whom R/S is a key component of mental and emotional well-being.

### Electronic supplementary material

Below is the link to the electronic supplementary material.

Additional File 1. PDF (.pdf). Mental Health Care Professional Survey. Survey used to collect mental health professionals’ views on the relevance of religion and spirituality to mental health care.

Additional File 2. PDF (.pdf). Table 1: Sample Demographic Characteristics. Frequency analysis on sample demographic charactistics.

Additional File 3. PDF (.pdf). Table 2: Sample Professional Background and Training. Frequency analysis on professional background and training of the sample.

Additional File 4. PDF (.pdf). Table 3: R/S Backgrounds, Beliefs, and Practices. Frequency analysis on sample spiritual and religious background, beliefs, and practices.

Additional File 5. PDF (.pdf). Table 4: Perceived Importance of Training in R/S Competencies. Descriptive analysis on sample perceived importance of training in R/S competencies.

Additional File 6. PDF (.pdf). Table 5: Explicit Training in Specific R/S Competencies. Descriptive analysis on sample explicit training in specific R/S competencies.

Additional File 7. PDF (.pdf). Table 6: Self-Rated R/S Competence. Descriptive analysis on sample self-rated R/S competencies.

## Data Availability

The datasets used and/or analyzed during the current study are available from the corresponding author on reasonable request.

## List of Abbreviations

SRBBPs: Spiritual and religious backgrounds, beliefs, and practices
R/S: Religious and spiritual
PTSD: Posttraumatic stress disorder
RSIPAS: Religious/Spiritually Integrated Practice Assessment Scale
DUREL: Duke University Religious Index
BMMRS: Brief Multidimensional Measure of Religiousness/Spirituality
MFT: Marriage and family therapy
LCSW: Licensed clinical social worker
IR: Intrinsic religiosity
