# Supplementary material for: Mental health professionals’ perspectives on the relevance of religion and spirituality to mental health care
Source: BMC Psychol. 2023 Dec 12;11:439. doi: 10.1186/s40359-023-01466-y (PMC10717464; doi:10.1186/s40359-023-01466-y)
Supplement: Supplementary file 1 — Additional File 1. PDF (.pdf). Mental Health Care Professional Survey. Survey used to collect mental health professionals’ views on the relevance of religion and spirituality to mental health care. [file 40359_2023_1466_MOESM1_ESM.pdf]

Supplementary Table 1. Frequency analysis on sample demographic characteristics

	n	%
**Age**		
25-34 years	76	8.5
35-44 years	179	20.1
45-54 years	223	25.0
55-64 years	235	26.4
65-74 years	141	15.8
75 + years	37	4.2
**Gender**		
Male	196	21.9
Female	697	78.0
Other	1	0.1
**Race/Ethnicity**		
White or Caucasian (not Hispanic)	712	79.6
Hispanic or Latina/Latino	105	11.7
African American/Black (not Hispanic)	58	6.5
Asian/Pacific Islander	13	1.5
American Indian or Alaskan Native	6	0.7
**Relationship Status**		
In a relationship (non-cohabiting)	23	2.6
Living with a partner	67	7.5
Never married	88	9.9
Married	542	61.0
Separated	11	1.2
Divorced	139	15.6
Widowed	19	2.1
**People in household (incl. oneself)**		
1	186	20.9
2	393	44.2
3	130	14.6
4	120	13.5
5	39	4.4

6	13	1.5
7 or more	9	1.0
**Income**		
Up to $19,999	6	0.7
$20,000 - $39,000	41	4.6
$40,000 - $59,000	106	11.9
$60,000 - $79,000	155	17.4
$80,000 - $99,000	132	14.8
$100,000 - $119,000	118	13.3
$120,000 - $139,000	71	8.0
$140,000 - $159,000	55	6.2
$160,000 - $179,000	23	2.6
$180,000 - $199,000	30	3.4
$200,000 - $299,000	52	5.8
Over $300,000	39	4.4
Decline to State	61	6.9
**Setting**		
Urban	241	27.0
Suburban	547	61.3
Rural	104	11.7
