# Supplementary material for: Mental health professionals’ perspectives on the relevance of religion and spirituality to mental health care
Source: BMC Psychol. 2023 Dec 12;11:439. doi: 10.1186/s40359-023-01466-y (PMC10717464; doi:10.1186/s40359-023-01466-y)
Supplement: Supplementary file 2 — Additional File 2. PDF (.pdf). Table 1: Sample Demographic Characteristics. Frequency analysis on sample demographic charactistics. [file 40359_2023_1466_MOESM2_ESM.pdf]

Thank you for considering our survey for Mental Health Care Professionals!

* 1. First, let's see if you are eligible to participate!

Are you a licensed or certified mental health provider currently providing mental health care to patients or clients?

- ◯ Yes
- ◯ No

* 2. What type(s) of mental health provider are you?

- ☐ Social Worker (LCSW or LMFW)
- ☐ Psychologist (Licensed Clinical Psychologist)
- ☐ Marriage Family Therapist (Licensed MFT)
- ☐ Psychiatrist (MD)
- ☐ Psychiatric Mental Health Nurse (PMH-RN)
- ☐ Other (please specify)
- ☐ Professional Counselor (LPC)
- ☐ Chemical Dependency Counselor (CAADAC)
- ☐ Pastoral Counselor (Certified or Licensed)
- ☐ None

* 3. How did you hear about this survey?

You are Eligible! Now we will ask you to Consent to Participate in this Research.

* 4. TITLE: Fostering spiritual and religious competencies in mental health care

Thank you for considering completing this survey!

This survey is part of a study asking for your expert opinion on how mental health care should deal with religious and spiritual issues. You'll be asked to complete a survey and a computerized reaction time task.

This study is being conducted by Cassandra Vieten at the Institute of Noetic Sciences in collaboration with Kenneth Pargament at Bowling Green State University, Michelle Pearce at the University of Maryland, and Holly Oxhandler at Baylor University, and is funded by the John Templeton Foundation.

If you participate, your responses will be collected anonymously. At the end of the survey, you may choose to enter your name and contact information in a completely separate database to be entered into a drawing or receive results of the study. Data collected will not be associated with your name or IP address. Individual results will not be analyzed or disseminated, and reports will not include any identifying information.

For your participation, you will be entered into a drawing to win one of four free iPads. The odds of having your name drawn are about 1 in 300. Your participation is completely voluntary. You do not have to join the study. Even if you decide to join now, you can change your mind later. You won't receive any direct benefit from being in this study, nor will it cost you to be part of the study.

If you participate, you will be asked to complete an online survey which will take approximately 25 minutes. Please only participate if you have that much time available and intend to complete the survey and reaction time task.

There is a small risk of breach of confidentiality. There is a small risk of being bored or being uncomfortable with some of the questions.

- ◯ Yes, I agree to participate in this research
- ◯ No thanks, I do not agree to participate in this research

OK, let's get started with some basic information about yourself.

5. Your age (at last birthday): ______ years

[ ]

* 6. Your gender:

- ○ Male
- ○ Female
- ○ Other (please specify)

[ ]

* 7. Your race/ethnicity:

- ○ White or Caucasian (not Hispanic)
- ○ American Indian or Alaskan Native
- ○ African American/Black (not Hispanic)
- ○ Asian/Pacific Islander
- ○ Hispanic or Latina/Latino

Multiple Ethnicities/Other (Please specify):

[ ]

8. Your relationship status (check the one that best describes you now):

- ○ Married
- ○ Widowed
- ○ Living with a Partner
- ○ Never Married
- ○ Separated
- ○ In a relationship with someone who does not live with me
- ○ Divorced

9. Last year, what was your approximate total family income?

- ○ Up to $19,999
- ○ $140,000 - $159,000
- ○ $20,000 - $39,000
- ○ $160,000 - $179,000
- ○ $40,000 - $59,000
- ○ $180,000 - $199,000
- ○ $60,000 - $79,000
- ○ $200,000 - $299,000
- ○ $80,000 - $99,000
- ○ Over $300,000
- ○ $100,000 - $119,000
- ○ Decline to State
- ○ $120,000 - $139,000

10. How many people (adults and children) live in your household? Please include yourself.

- ◯ 1
- ◯ 2
- ◯ 3
- ◯ 4
- ◯ 5
- ◯ 6
- ◯ 7 or more

11. What state do you live in?

I live in a different country (if so, which one?)

12. In what type of setting do you live?

- ◯ Urban
- ◯ Suburban
- ◯ Rural

Now, some questions about your training and practice...

13. What is your highest level of education completed?

- ○ Some High School
- ○ GED
- ○ High School Graduate
- ○ Some College
- ○ Associate's Degree
- ○ Bachelor's Graduate
- ○ Master's Degree
- ○ Doctoral Degree (e.g., M.D., Ph.D.)

Other (please specify)

14. In what year did you receive your last completed degree?

15. What license(s) do you currently hold to practice (Please check all that apply)

- ☐ APN (Advanced Practice Nurse)
- ☐ LCSW (Licensed Clinical Social Worker)
- ☐ LMSW (Licensed Master in Social Worker)
- ☐ LPC (Licensed Professional Counselor)
- ☐ LMFT (Licensed Marriage and Family Therapist)
- ☐ LCDC (Licensed Chemical Dependence Counselor)
- ☐ PhD (Doctor of Philosophy)
- ☐ PsyD (Doctor of Psychology)
- ☐ EdD (Doctor of Education)
- ☐ MD (Doctor of Medicine)
- ☐ Other (please specify)

16. For how many years have you been in clinical practice (providing psychotherapy or mental health care to clients/patients), including internship and residency?

17. Which age groups do you regularly work with in your practice (Check all that apply):

- ☐ Infants/Toddlers (0-3)
- ☐ Children (4-12)
- ☐ Adolescents (13-17)
- ☐ Young adults (18-29)
- ☐ Adults (30-45)
- ☐ Middle-aged adults (46-64)
- ☐ Older adults (65+)

18. How many client-hours on average do you currently spend providing mental health services to clients or patients each week?

- ○ Less than 10
- ○ 11-20
- ○ 21-30
- ○ 31-40
- ○ Over 40

19. What clinical issues do you deal with regularly in your practice (Check all that apply):

- ☐ Addiction
- ☐ Aging
- ☐ Child
- ☐ Adolescent/Young Adult
- ☐ End of Life/Palliative Care
- ☐ Severe Mental Illness/Psychosis
- ☐ Health/Medical care
- ☐ LGBTQ
- ☐ Other (please specify)
- ☐ Marital/Family/Couples
- ☐ Women's Issues
- ☐ Trauma
- ☐ Depression/Anxiety/Mood Disorders
- ☐ School-related
- ☐ Personality Disorders
- ☐ Criminal Justice & Juvenile Justice

20. How would you describe your current primary employment/practice setting? (check all that apply)

- ☐ Non-profit agency
- ☐ For-profit agency
- ☐ Public/private teaching hospital
- ☐ Public/private non-teaching hospital
- ☐ Child protective services
- ☐ Public welfare
- ☐ Solo private practice
- ☐ Group private practice
- ☐ Governmental agency
- ☐ Other (please specify)
- ☐ Mental health services
- ☐ Managed care
- ☐ School (K-12)
- ☐ School (college/university)
- ☐ Judicial system
- ☐ Currently unemployed
- ☐ Currently enrolled in school
- ☐ Retired

21. Please rate how much proficiency you have in attending to or integrating your clients' or patients' spiritual and religious backgrounds, beliefs and practices in mental health care?

- ○ None
- ○ Not very much
- ○ Some
- ○ Quite a bit
- ○ A lot

22. When you were a student in your professional degree program, did you ever receive any education on religion and/or spirituality as a form of multicultural diversity that you might encounter in your clients (similar to age, gender, ethnicity, or sexual orientation)?

- ○ None
- ○ Not very much
- ○ Some
- ○ Quite a bit
- ○ A lot

23. When you were a student in your degree program, did you ever take any courses that focused primarily on ways to address or include clients' religion/spirituality in your clinical practice?

- ○ Yes – If yes, enter the number of courses below
- ○ No course, but received some information
- ○ No course, did not receive any information

If yes, how many courses?

[ ]

24. Have you ever taken any continuing education workshops or courses that focused primarily on attending to religion/spirituality in clinical practice (not including courses taken as a student before earning your professional degree)?

- ○ Yes (if yes, enter how many in the space below)
- ○ No

If yes, how many workshops or courses?

[ ]

25. Have you gained any other experience or training in attending to religion/spirituality in clinical practice? (check all that apply)

- ☐ Reading books or articles
- ☐ Attending conference presentations
- ☐ Clinical Supervision/Consultation
- ☐ Other (please specify)
- ☐ Conversations
- ☐ Retreats
- ☐ Personal Exploration

[ ]

Here are some working definitions...

Throughout this survey, you will be asked about your views related to religion, spirituality, and mental health. To help you with answering these questions, here are a few definitions for each of these terms:

Religion **is defined here as "the search for significance that occurs within the context of established institutions that are primarily designed to facilitate spirituality" (Pargament et al., 2013) through a shared set of beliefs and practices.**

Spirituality **is defined more broadly as "a search for the sacred" (Pargament et al., 2013). The sacred includes not only different understandings of God or the divine, but also aspects of life that are experienced as divine-like, or transcending the self, such as nature, love, or beauty.**

Some people identify themselves as religious, some identify themselves as spiritual but not religious, and others as both spiritual and religious.

We will also be asking about mental health and mental illness.

We define *mental health* as a state of well-being in which every individual works to realize his or her potential, can cope with the normal stresses of life, can work productively and fruitfully, and is able to make a contribution to her or his community (World Health Organization, 2014).

We define *mental illness*, or mental health problems, as significant difficulties in one's thinking, emotion, and/or behavior that result in being unable to cope with the normal stressors of life, work productively and fruitfully, or make contributions to his/her community

Now, some questions about your views...

26. In general, how important do you believe discussion of religious/spiritual issues is to therapy?

- ◯ Not At All
- ◯ Not Much
- ◯ Uncertain
- ◯ Somewhat
- ◯ Very Much

27. In order to resolve the concerns that bring your clients into therapy, how important do you think it is for them to be able to discuss religious/spiritual issues with you?

- ◯ Not At All
- ◯ Not Much
- ◯ Uncertain
- ◯ Somewhat
- ◯ Very Much

28. How much do you think your clients would like to discuss religious/spiritual issues with you?

- ◯ Not At All
- ◯ Not Much
- ◯ Uncertain
- ◯ Somewhat
- ◯ Very Much

29. I think I should wait for my clients to bring up religion/spirituality, rather than my bringing it up.

- ◯ Strongly Disagree
- ◯ Disagree
- ◯ Neutral
- ◯ Agree
- ◯ Strongly Agree

30. In general, how willing are you to discuss religious/spiritual issues with your patients or clients?

- ◯ Not At All
- ◯ Not Much
- ◯ Uncertain
- ◯ Somewhat
- ◯ Very Much

31. Do any of the following make it less likely that you would attend to spirituality or religion in taking a history, assessment/diagnosis, treatment planning, psychotherapeutic interventions or referrals? Check all that apply:

- [ ] Not enough time
- [ ] My institution or setting does not support it
- [ ] I don’t think it is important
- [ ] I don’t have enough training in it
- [ ] I don’t think my clients or patients would appreciate it
- [ ] I don’t think religious or spiritual issues should be discussed in clinical work
- [ ] I feel personally uncomfortable doing so
- [ ] Nothing makes it less likely
- [ ] Other (please specify)

32. To what extent do you agree or disagree with these statements?

	Strongly Disagree	Disagree	Neutral	Agree	Strongly Agree
Engaging in religious/spiritual practices (e.g., prayer, religious services, reading religious texts) improves mental health.	○	○	○	○	○
I consider religion/spirituality to be relevant to mental health.	○	○	○	○	○
There is a religious/spiritual dimension to people’s mental health.	○	○	○	○	○
I believe people have a higher power (e.g., God) who wants them to experience mental health.	○	○	○	○	○

33. In what percentage of your clients/patients do you estimate that you have verbally inquired about religion or spirituality in the course of assessment or treatment?

34. Please indicate the response to the right that best fits how much you agree or disagree with these statements:

	Strongly Disagree	Disagree	Neutral	Agree	Strongly Agree
It is essential to assess clients’ religious/spiritual beliefs in practice.	○	○	○	○	○
I consider my clients' religion/spirituality to be relevant to their mental health.	○	○	○	○	○
I do my best to help clients find coping skills that do *not* rely on their religious or spiritual beliefs.	○	○	○	○	○
There is a religious/spiritual dimension to my clients’ mental health.	○	○	○	○	○
Integrating clients’ religious/spiritual needs during treatment helps improve client outcomes.	○	○	○	○	○
Bringing up religious/spiritual issues during treatment violates client privacy.	○	○	○	○	○
Engaging in religious/spiritual practices (e.g., prayer, religious services, reading religious texts) generally seems to improve my clients’ mental health.	○	○	○	○	○
It is part of my job as a clinician to help clients find a spiritual or religious path.	○	○	○	○	○
Addressing religious/spiritual issues in a public hospital or clinic violates the separation of church and state.	○	○	○	○	○

	Strongly Disagree	Disagree	Neutral	Agree	Strongly Agree
I am open to referring my clients to spiritual, religious or pastoral counseling.	○	○	○	○	○
My clients' mental illness or symptoms can improve without considering their religion/spirituality.	○	○	○	○	○
Attending to clients' religious/spiritual beliefs is consistent with my profession's code of ethics.	○	○	○	○	○
Some religious and spiritual beliefs are not healthy for people's mental, emotional, or psychological well-being.	○	○	○	○	○
Engaging in religious/spiritual practices (e.g., prayer, religious services, reading religious texts) seems to help my clients cope with symptoms of mental illness.	○	○	○	○	○
Empirically-supported religious/spiritually integrated treatments are relevant to my practice.	○	○	○	○	○
There is a religious/spiritual dimension to the work I do.	○	○	○	○	○
I refuse to work within my clients' religious/spiritual belief system if it differs from my own.	○	○	○	○	○

Now we want your thoughts on training and competence in the mental health field overall.

What we mean by spiritual and religious competencies is the set of attitudes, knowledge and skills in the domains of spirituality and religion (if any) that you believe ALL mental health care providers (not just specialists) should expect to receive training in and demonstrate. These would appear on licensing exams for example, or in basic coursework or textbooks.

Attitudes refer to the implicit and explicit perspectives and biases mental health care providers hold about spirituality and religion as it relates to clinical practice.

Knowledge refers to information, facts, concepts, and awareness of research literature mental health care providers possess about spirituality and religion as it relates to clinical practice.

Skills refer to mental health care providers' use of their knowledge of religion and spirituality in their clinical work with clients.

In the next section, you will be asked to respond to sixteen potential competencies that could serve as guidelines for training mental health professionals. Let us know what you think.

35. Please answer the questions about the extent to which you see each area as important for mental health professionals, and your training in each area.

	Do you believe that mental health care providers should receive explicit training in this area?	In my training program (formal coursework or specific training during internship or residency):	I am able to do this in my clinical practice:
Demonstrating empathy, respect, and appreciation for clients from diverse spiritual, religious or secular backgrounds and affiliations.			
Viewing spirituality and religion as important aspects of human diversity, along with factors such as race, ethnicity, sexual orientation, socioeconomic status, disability, gender, and age.			
Being aware of how as clinicians, their **own** spiritual and/or religious background and beliefs may influence their clinical practice, and their attitudes, perceptions, and assumptions about the nature of psychological processes.			
Knowing that there are many diverse forms of spirituality and/or religion, and being willing to learn about spiritual and/or religious beliefs, communities, and practices that are important to their clients.			
Being able to describe how spirituality and religion can be viewed as overlapping, yet distinct, constructs.			

	Do you believe that mental health care providers should receive explicit training in this area?	In my training program (formal coursework or specific training during internship or residency):	I am able to do this in my clinical practice:
Being able to describe ways that clients may have experiences that are consistent with their spirituality or religion, yet may be difficult to differentiate from psychopathological symptoms.			
Knowing some ways that spiritual and/or religious beliefs, practices and experiences can develop and change over the lifespan.			
Being aware of spiritual and/or religious resources and practices that research indicates may support psychological wellbeing, and recovery from psychological disorders.			
Being able to identify spiritual and religious experiences, practices and beliefs that may have the potential to *negatively* impact mental health.			
Being aware of legal and ethical issues related to spirituality and/or religion that may surface when working with clients.			
Being able to conduct empathic and effective psychotherapy with clients from diverse spiritual and/or religious backgrounds, affiliations, and levels of involvement.			

	Do you believe that mental health care providers should receive explicit training in this area?	In my training program (formal coursework or specific training during internship or residency):	I am able to do this in my clinical practice:
Being able to inquire about spiritual and/or religious background, experience, practices, attitudes and beliefs as a standard part of understanding a client's history.			
Knowing how to help clients explore and access their spiritual and/or religious strengths and resources.			
Being able to identify and address spiritual and/or religious problems in clinical practice.			
Recognizing the limits of their qualifications and competence in the spiritual and/or religious domains, including their responses to clients spirituality and/or religion that may interfere with clinical practice, and being willing to 1) seek consultation from and collaborate with other qualified clinicians or spiritual/religious leaders (e.g. priests, pastors, rabbis, imam, spiritual teachers, etc.), 2) seek further training and education, and/or 3) refer appropriate clients to more qualified individuals and resources.			

36. If you wish, please comment on any of the proposed areas of competence (e.g. do you have any other responses to these areas of competence, or suggestions regarding the wording of these competencies?)

Whew! You are getting close to being done.

37. Please indicate how frequently you ACTUALLY have done each of the following in your clinical practice. No response is good or bad in terms of your own competence, we are just interested in how frequently these things occur in the real world of clinical practice.

	Never	Rarely	Some of the Time	Often	Very Often
Seek out consultation on how to address clients' religious/spiritual issues in treatment.	○	○	○	○	○
Read about ways to integrate clients' religion/spirituality to guide my practice decisions.	○	○	○	○	○
Read about research evidence on religion/spirituality and its relationship to health to guide my practice decisions.	○	○	○	○	○
Involve clients in deciding whether their religion/spirituality should be integrated into their treatment.	○	○	○	○	○
Use empirically supported interventions that specifically outline how to integrate my clients' religion/spirituality into treatment.	○	○	○	○	○
Conduct a full bio-psycho-social-spiritual assessment with each of my clients.	○	○	○	○	○
Link clients with religious/spiritual resources when it may potentially help them (e.g. religious/spiritual reading materials, contact information to local clergy, or a prayer room/place of worship).	○	○	○	○	○

	Never	Rarely	Some of the Time	Often	Very Often
Help clients consider ways their religious/spiritual support systems may be helpful.	○	○	○	○	○
Help clients consider the religious/spiritual meaning and purpose of their current life situations.	○	○	○	○	○

38. Is there anything that might keep you from**inquiring or asking** clients or patients about their religion or spirituality and how it is related to their psychological or emotional well-being?

39. Is there anything that has hindered, prevented, or discouraged you from**including or integrating** your clients' religious and/or spiritual beliefs in your clinical practice?

40. Is there anything that has helped or supported you to assess and/or integrate your clients' religious and/or spiritual beliefs in your clinical practice?

41. Is there anything you have done regarding assessing or including your clients' religion and/or spirituality in mental health treatment that you have found to be particularly helpful or supportive?

OK - last questions! These are about your own experience with religion and/or spirituality.

42. What is your religious preference?

- ○ Protestant
- ○ Hinduism
- ○ Catholic
- ○ Buddhism
- ○ Jewish
- ○ Muslim
- ○ None
- ○ Spiritual but not Religious

Other (please specify)

____

43. (If none, skip this question) I would consider my religious or spiritual beliefs to generally be:

- ☐ Extremely Liberal/Alternative
- ☐ Liberal
- ☐ Slightly Liberal
- ☐ Moderate
- ☐ Slightly Conservative
- ☐ Conservative
- ☐ Extremely Conservative/Traditional

44. To what extent do you consider yourself a religious person?

- ○ Not Religious
- ○ Slightly Religious
- ○ Moderately Religious
- ○ Very Religious

45. To what extent do you consider yourself a spiritual person?

- ○ Not Spiritual
- ○ Slightly Spiritual
- ○ Moderately Spiritual
- ○ Very Spiritual

46. How much did religion or spirituality influence your upbringing (in other words, how much was religion or spirituality a part of your family life while growing up?

- ○ Not at all
- ○ A little bit
- ○ Somewhat
- ○ A fair amount
- ○ Quite a bit
- ○ Very much

47. Below are some common methods of religious/spiritual practice. Which of these do you engage in regularly? (Please check all that apply).

- ☐ Attending religious service
- ☐ Attending small social gatherings on a religious/spiritual matter (e.g. Bible studies, spiritual discussion group)
- ☐ Listening to religious/spiritual music or radio
- ☐ Prayer
- ☐ Meditation
- ☐ Other (please specify)
- ☐ Reading religious or spiritual texts
- ☐ Watching religious/spiritual TV or videos
- ☐ Worship (outside of a religious service)
- ☐ Yoga or some other form of physical practice (please specify)
- ☐ None of the above

48. How often do you attend formal religious or spiritual services, rituals, or gatherings?

- ○ Never
- ○ Once a year or less
- ○ A few times a year
- ○ A few times a month
- ○ Once a week
- ○ More than once a week

49. How often do you spend time in private religious or spiritual activities, such as prayer, meditation or study of religious or spiritual teachings or texts?

- ○ Rarely or never
- ○ A few times a month
- ○ Once a week
- ○ Two or more times a week
- ○ Daily
- ○ More than once a day

The following section contains 3 statements about religious belief or experience. Please mark the extent to which each statement is true or not true for you.

50. In my life, I experience the presence of the Divine (i.e., God).

- ○ Definitely *not* true
- ○ Tends *not* to be true
- ○ Unsure
- ○ Tends to be true
- ○ Definitely true

51. My religious or spiritual beliefs are what really lie behind my whole approach to life.

- ○ Definitely *not* true
- ○ Tends *not* to be true
- ○ Unsure
- ○ Tends to be true
- ○ Definitely true

52. I try hard to carry my religion or spirituality over into all other dealings in life.

- Definitely *not* true
- Tends *not* to be true
- Unsure
- Tends to be true
- Definitely true

Reaction Time Task

53. Thank you for completing the survey! You are almost done!!

Are you at a desktop or laptop computer with a keyboard?

- ◯ Yes
- ◯ No

54. If you are on a phone or tablet, do you currently have access to a laptop or desktop computer?

- ◯ Yes (please go to that laptop/desktop now and bring your phone/tablet with you)
- ◯ No

Create Your Own Study ID

* 55. OK, before you move on to the final task on your desktop or laptop computer, please create an ID number for yourself that has any two letters, two numbers, and then two letters again (for example, the first two letters of your first name, the first two numbers of your address, and the first two letters of the city you live in).

Your ID should look something like this: **CA47NO** or **AR81SE**

Please create an ID now, and write it down, because you will be asked to enter it on your desktop or laptop.

Reaction Time Task

OK, now we'd like for you to complete a reaction time task.

In the reaction time task, you'll be asked to sort words and pair words together as quickly as possible. PLEASE MAKE SURE YOU ARE IN A PLACE WHERE YOU CAN FOCUS ON THE TASK UNINTERRUPTED FOR ABOUT 8 MINUTES. Please turn off your phone and close your door.

If you were already on a laptop or desktop with a full keyboard,please click this link, which will open a new window or tab in your browser containing a reaction time task. Once you are finished with the task, please return to this page to enter the drawing.

If you just switched from a tablet or phone to laptop/desktop with a full keyboard,type in the URL below into your browser:
https://tinyurl.com/MH-Survey-IPAD
which will open a new window or tab in your browser containing a reaction time task. Once you are finished with the task, please return to this page on your tablet or phone to enter the drawing.

Please contact nfry@noetic.org if you have any trouble.

56. Did you complete the Reaction Time Task?

- ◯ Yes (I completed both the survey and the reaction time task)
- ◯ No, I was unable to complete the Reaction Time Task (please explain below)

I was unable to complete the Reaction Time Task because (please specify)...

[ ]

iPad Drawing

Thank you for completing this study! Your responses are anonymous, meaning that we have not collected any personal information about you as you have completed this survey (including your IP address).

Please click **here** to enter your contact information if you would like to be entered into the drawing to **win a free iPad**, receive a summary of the results of this study, or **learn about another study that offers free CE training for those who qualify.**

(If that link does not work, please copy and paste this URL into your browser: https://www.research.net/r/MHiPodRaffle)
