# Supplementary material for: Mental health professionals’ perspectives on the relevance of religion and spirituality to mental health care
Source: BMC Psychol. 2023 Dec 12;11:439. doi: 10.1186/s40359-023-01466-y (PMC10717464; doi:10.1186/s40359-023-01466-y)
Supplement: Supplementary file 3 — Additional File 3. PDF (.pdf). Table 2: Sample Professional Background and Training. Frequency analysis on professional background and training of the sample. [file 40359_2023_1466_MOESM3_ESM.pdf]

Supplementary Table 2. Frequency Analysis on Professional Background and Training of the Sample

	n	%
**Degree**		
Some college	1	.1
Bachelor's graduate	8	.9
Master's degree	597	67.6
Doctoral degree	277	31.4
**License**		
Licensed social worker (LCSW/LMFW)	254	28.4
Licensed clinical psychologist	129	14.4
Licensed marriage family therapist (MFT)	171	19.1
Psychiatrist (MD)	39	4.4
Psychiatric mental health nurse (PMH-RN)	7	0.8
Professional counselor (LPC)	196	21.9
Chemical dependency counselor (CAADAC)	18	2.0
**Client hours per week**		
Less than 10 hours	158	17.9
11-20 hours	122	13.8
21-30 hours	260	29.4
31-40 hours	152	17.2
41-50 hours	140	15.8
Over 50+ hours	53	6.0
**Practice setting**		
Solo private practice	359	40.2
Non-profit agency	184	20.6
Mental health services	151	16.9
Group private practice	128	14.3
For-profit agency/Managed care	117	13.1
Public welfare/Governmental agency/Judicial system	90	10.1
School (K-12)/School (College/university)	68	7.6
Public/private hospital (teaching/non-teaching)	61	6.8
Student/Not Employed/Retired	26	2.9
