# Supplementary material for: Mental health professionals’ perspectives on the relevance of religion and spirituality to mental health care
Source: BMC Psychol. 2023 Dec 12;11:439. doi: 10.1186/s40359-023-01466-y (PMC10717464; doi:10.1186/s40359-023-01466-y)
Supplement: Supplementary file 4 — Additional File 4. PDF (.pdf). Table 3: R/S Backgrounds, Beliefs, and Practices. Frequency analysis on sample spiritual and religious background, beliefs, and practices. [file 40359_2023_1466_MOESM4_ESM.pdf]

Supplementary Table 4. Descriptive analysis on perceived importance of training in R/S competencies

	M	*SD*	% Very much	% Somewhat	% Very much + somewhat
**Do you believe that mental health care providers should receive explicit training in this area?**					
1. Empathy, respect, and appreciation to R/S diverse clients	3.75	0.53	79.3	16.7	96.0
2. Empathic and effective psychotherapy with R/S diverse clients	3.75	0.57	80.5	14.4	94.9
3. Awareness of clinicians' R/S influence on psychological processes	3.72	0.60	78.6	16.2	94.8
4. Understanding of R/S importance to human diversity	3.71	0.59	77.5	16.7	94.2
5. Knowledge of R/S diverse forms	3.68	0.61	74.1	20.2	94.3
6. Recognition of R/S knowledge limits and willingness for further education	3.60	0.74	72.2	17.9	90.1
7. Ability to inquire about R/S issues	3.59	0.69	69.3	22.8	92.1
8. Awareness of R/S legal and ethical issues related to clinical practice	3.47	0.86	65.6	21.3	86.9
9. Knowledge of R/S lifespan development	3.44	0.79	59.8	27.6	87.4

10. Identification of potentially harmful R/S practice, beliefs, experiences	3.42	0.84	60.6	25.1	85.7
11. Differentiation between R/S experiences and psychopathological symptoms	3.40	0.82	57.2	29.1	86.3
12. Awareness of R/S resources/practices supporting mental health	3.39	0.83	58.0	27.2	85.2
13. Differentiation between spirituality and religion	3.35	0.85	55.1	29.7	84.8
14. Helping clients explore and access R/S strengths and resources	3.31	0.88	53.9	29.1	83.0
15. Identification and address of R/S problems in clinical practice	3.27	0.89	51.4	29.8	81.2
**Across Items**	3.52	0.74			

Note. Items appear in descending order based on mean scores. Item values correspond to 1 = Not at all, 2 = A little, 3 = Somewhat, 4 = Very much
