# Supplementary material for: Mental health professionals’ perspectives on the relevance of religion and spirituality to mental health care
Source: BMC Psychol. 2023 Dec 12;11:439. doi: 10.1186/s40359-023-01466-y (PMC10717464; doi:10.1186/s40359-023-01466-y)
Supplement: Supplementary file 5 — Additional File 5. PDF (.pdf). Table 4: Perceived Importance of Training in R/S Competencies. Descriptive analysis on sample perceived importance of training in R/S competencies. [file 40359_2023_1466_MOESM5_ESM.pdf]

Supplementary Table 3. Frequency Analysis on Spiritual and Religious Background, Beliefs and Practices

	n	%
**Religious preference**		
Protestant	189	24.7
Catholic	126	16.4
Buddhism	35	4.6
Jewish	70	9.1
Hinduism	4	0.5
Muslim	7	0.9
Spiritual but not religious	269	35.1
None	66	8.6
**Religious beliefs**		
Extremely liberal/alternative	130	14.5
Liberal	286	32.0
Slightly liberal	84	9.4
Moderate	152	17.0
Slightly conservative	68	7.6
Conservative	69	7.7
Extremely conservative/traditional	21	2.3
**Religious orientation**		
Not religious	319	35.9
Slightly religious	247	27.8
Moderately religious	218	24.5
Very religious	105	11.8
**Spiritual orientation**		
Not spiritual	38	4.3
Slightly Spiritual	135	15.2
Moderately Spiritual	286	32.1
Very Spiritual	431	48.4
**Religious upbringing**		
Not at all	56	6.3
A little bit	124	13.9
Somewhat	143	16.0
A fair amount	164	18.3

Quite a bit	182	20.4
Very much	225	25.2
**R/S gatherings attendance**		
Never	162	18.1
Once a year or less	160	17.9
A few times a year	258	28.9
A few times a month	111	12.4
Once a week	143	16.0
More than once a week	59	6.6
**Private R/S activities**		
Rarely or never	191	21.4
A few times a month	134	15.0
Once a week	53	5.9
Two or more times a week	167	18.7
Daily	264	29.6
More than once a day	82	9.2
**Experience of divine presence**		
Definitely not true	82	9.2
Not true	61	6.9
Unsure	107	12.0
True	187	21.0
Definitely true	452	50.8
**R/S approach to life**		
Definitely not true	107	12.0
Not true	61	6.8
Unsure	84	9.4
True	289	32.4
Definitely true	351	39.3
**R/S consideration into life aspects**		
Definitely not true	117	13.1
Not true	104	11.6
Unsure	89	10.0
True	293	32.8
Definitely true	291	32.6
