# Supplementary material for: Mental health professionals’ perspectives on the relevance of religion and spirituality to mental health care
Source: BMC Psychol. 2023 Dec 12;11:439. doi: 10.1186/s40359-023-01466-y (PMC10717464; doi:10.1186/s40359-023-01466-y)
Supplement: Supplementary file 6 — Additional File 6. PDF (.pdf). Table 5: Explicit Training in Specific R/S Competencies. Descriptive analysis on sample explicit training in specific R/S competencies. [file 40359_2023_1466_MOESM6_ESM.pdf]

Supplementary Table 5. Descriptive analysis on explicit training in specific R/S competencies

	M	*SD*	% No training	% A little bit	No training + a little bit
**R/S training during training program**					
1. Identification of potentially harmful R/S practice, beliefs, experiences	1.64	0.83	54.9	29.3	84.2
2. Awareness of R/S resources/practices supporting mental health	1.72	0.84	50.0	31.5	81.5
3. Identification and address of R/S problems in clinical practice	1.72	0.86	51.3	29.6	80.9
4. Knowledge of R/S lifespan development	1.76	0.89	49.9	28.8	78.7
5. Awareness of R/S legal and ethical issues related to clinical practice	1.78	0.93	50.3	27.3	77.6
6. Differentiation between spirituality and religion	1.80	0.91	47.9	28.9	76.8
7. Helping clients explore and access R/S strengths and resources	1.82	0.90	46.5	30.0	76.5
8. Differentiation between R/S experiences and psychopathological symptoms	1.92	0.88	38.3	37.1	75.4
9. Recognition of R/S knowledge limits and willingness for further education	2.00	0.98	39.4	30.0	69.4
10. Knowledge of R/S diverse forms	2.17	0.93	27.8	34.9	62.7
11. Ability to inquire about R/S issues	2.28	1.01	26.2	34.1	60.3
12. Empathy, respect, and appreciation to R/S diverse clients	2.29	1.00	26.7	30.4	57.1
13. Understanding of R/S importance to human diversity	2.36	0.97	22.5	31.4	53.9
14. Empathic and effective psychotherapy with R/S diverse clients	2.39	0.99	22.2	31.3	53.6

15. Awareness of clinicians' R/S influence on psychological processes	2.42	1.01	22.7	29.5	52.2

Note. Items appear in ascending order based on mean scores. Item values correspond to 1 = No training, 2 = A little bit of training, 3 = Some training, 4 = Comprehensive formal training
