# Supplementary material for: Mental health professionals’ perspectives on the relevance of religion and spirituality to mental health care
Source: BMC Psychol. 2023 Dec 12;11:439. doi: 10.1186/s40359-023-01466-y (PMC10717464; doi:10.1186/s40359-023-01466-y)
Supplement: Supplementary file 7 — Additional File 7. PDF (.pdf). Table 6: Self-Rated R/S Competence. Descriptive analysis on sample self-rated R/S competencies. [file 40359_2023_1466_MOESM7_ESM.pdf]

Supplementary Table 6. Descriptive analysis on self-rated R/S competencies

	M	*SD*	% Very much	% Completely	% Very much + completely
**I am able to do this in my clinical practice:**					
1. Awareness of R/S legal and ethical issues related to clinical practice	3.15	1.15	22.9	14.1	37.0
2. Identification of potentially harmful R/S practice, beliefs, experiences	3.28	1.09	31.2	13.1	44.3
3. Identification and address of R/S problems in clinical practice	3.31	1.09	27.7	15.2	42.9
4. Awareness of R/S resources/practices supporting mental health	3.32	1.04	30.0	13.2	43.2
5. Helping clients explore and access R/S strengths and resources	3.46	1.07	30.5	18.6	49.1
6. Differentiation between R/S experiences and psychopathological symptoms	3.48	0.97	34.4	14.9	49.3
7. Knowledge of R/S lifespan development	3.51	1.02	33.3	17.8	51.1
8. Recognition of R/S knowledge limits and willingness for further education	3.64	1.06	30.3	25.2	55.5
9. Differentiation between spirituality and religion	3.69	1.01	36.7	23.2	59.9
10. Knowledge of R/S diverse forms	3.87	0.88	43.9	25.0	68.9
11. Empathic and effective psychotherapy with R/S diverse clients	3.95	0.87	45.0	27.8	72.8
12. Ability to inquire about R/S issues	3.96	0.98	36.2	34.4	70.6
13. Understanding of R/S importance to human diversity	3.99	0.85	43.7	30.0	73.7
14. Empathy, respect, and appreciation to R/S diverse clients	4.03	0.86	42.0	33.1	75.1

15. Awareness of clinicians' R/S influence on psychological processes	4.01	0.88	43.0	31.8	74.8

Note. Items appear in ascending order based on mean scores. Item values correspond to 1 = Not at all, 2 = Not much, 3 = Somewhat, 4 = Very much, 5 = Completely
